# Transcriptional responses of *Candida glabrata* biofilm cells to fluconazole are modulated by the carbon source

**DOI:** 10.1038/s41522-020-0114-5

**Published:** 2020-01-23

**Authors:** Rosana Alves, Stavroula L. Kastora, Alexandra Gomes-Gonçalves, Nuno Azevedo, Célia F. Rodrigues, Sónia Silva, Liesbeth Demuyser, Patrick Van Dijck, Margarida Casal, Alistair J. P. Brown, Mariana Henriques, Sandra Paiva

**Affiliations:** 1 Center of Molecular and Environmental Biology, Department of Biology, University of Minho, Braga, Portugal; 2 Aberdeen Fungal Group, University of Aberdeen, Institute of Medical Sciences, Foresterhill, Aberdeen, UK; 3 LIBRO - Laboratório de Investigação em Biofilmes Rosário Oliveira, Center for Biological Engineering, University of Minho, Braga, Portugal; 4 LEPABE, Department of Chemical Engineering, University of Porto, Porto, Portugal; 5 VIB-KU Leuven Center for Microbiology, Flanders, Belgium; 6 Laboratory of Molecular Cell Biology, Institute of Botany and Microbiology, KU Leuven, Leuven, Belgium; 7 MRC Center for Medical Mycology, University of Exeter, Geoffrey Pope Building, Stocker Road, Exeter, UK

**Keywords:** Antimicrobials, Next-generation sequencing, Pathogens, Biofilms, Cellular microbiology

## Abstract

*Candida glabrata* is an important human fungal pathogen known to trigger serious infections in immune-compromised individuals. Its ability to form biofilms, which exhibit high tolerance to antifungal treatments, has been considered as an important virulence factor. However, the mechanisms involving antifungal resistance in biofilms and the impact of host niche environments on these processes are still poorly defined. In this study, we performed a whole-transcriptome analysis of *C. glabrata* biofilm cells exposed to different environmental conditions and constraints in order to identify the molecular pathways involved in fluconazole resistance and understand how acidic pH niches, associated with the presence of acetic acid, are able to modulate these responses. We show that fluconazole treatment induces gene expression reprogramming in a carbon source and pH-dependent manner. This is particularly relevant for a set of genes involved in DNA replication, ergosterol, and ubiquinone biosynthesis. We also provide additional evidence that the loss of mitochondrial function is associated with fluconazole resistance, independently of the growth condition. Lastly, we propose that *C. glabrata* Mge1, a cochaperone involved in iron metabolism and protein import into the mitochondria, is a key regulator of fluconazole susceptibility during carbon and pH adaptation by reducing the metabolic flux towards toxic sterol formation. These new findings suggest that different host microenvironments influence directly the physiology of *C. glabrata*, with implications on how this pathogen responds to antifungal treatment. Our analyses identify several pathways that can be targeted and will potentially prove to be useful for developing new antifungals to treat biofilm-based infections.

## Introduction

*Candida spp*. are important fungal pathogens known to trigger serious infections in immune-compromised individuals, affecting billions of people every year.^1^ While *C. albicans* has been predominantly identified as the most common cause of candidiasis, infections caused by non-*albicans Candida* strains such as *C. glabrata* have been increasing worldwide.^2,3^ This trend has coincided with the prophylactic use of antifungals, which has resulted in increased drug resistance.^4^ Moreover, *C. glabrata* has the ability to adhere and to form biofilms on both biotic and abiotic surfaces, such as host tissues and implanted medical devices.^5–8^ This ability makes these infections a clinical challenge, as the cells in biofilms are also intrinsically resistant to conventional antifungal treatments.^9^

In order to survive and successfully proliferate in the different host niches, *C. glabrata* must rapidly adapt to a diverse range of environmental stresses, such as temperature, pH fluctuations, and nutrient availability. Some of these niches are complex, dynamic and frequently limited in the content of carbon sources available. To survive in such environments, these pathogens have to control the expression of key metabolic functions.^10–12^ For instance, during gastrointestinal and vaginal colonization, where glucose concentration is low, alternative carbon sources such as acetate or lactate are particularly abundant^13,14^ and may support the growth and the proliferation of *C. glabrata* cells. Interestingly, low-glucose environments were found to induce the formation of *C. glabrata* biofilms and confer resistance to antifungal treatment.^15^ This behavior suggests that this pathogen has the capacity to adjust its lifestyle in accordance to nutrient availability and determine the outcome of the next phase: either to continue as part of a biofilm population or disperse to find new colonization sites. However, little is known about the physiological effect of acidic environments, containing alternative non-fermentable carbon sources such as acetate, on the antifungal treatment of *C. glabrata* biofilms. Our group has previously demonstrated that *C. glabrata* cells were more susceptible to fluconazole and better phagocytosed and killed by macrophages, when exposed to both glucose and to physiological concentrations of acetate.^16^ Growth in the presence of this substrate also affected the ability of these cells to form biofilms.^16^ Although several putative acetate transporters and channels were identified to be involved in the response to acetate and fluconazole, in both planktonic and biofilm cells, the exact molecular mechanisms underlying fluconazole resistance in biofilms under acidic conditions are still unclear. In this work, the specific transcriptomic responses of *C. glabrata* biofilm cells to fluconazole, when grown in the presence of glucose or glucose and acetate, were evaluated by RNA sequencing. This allowed us to decipher the differentially expressed genes and potential mechanisms developed by biofilms to adapt to these different physiological environments.

Our data represent a global view of transcriptomic regulation in *C. glabrata* biofilms in response to carbon adaptation and fluconazole resistance. Considering the urgent need to find adequate and more effective therapeutic approaches to treat *Candida* infections, further understanding of the molecular mechanisms underlying biofilm formation and antifungal resistance could lead to the development of novel inhibitors to control the dissemination of these pathogens.

## Results

### Transcriptional responses of *C. glabrata* biofilm cells to fluconazole are modulated by the presence of acetate

*C. glabrata* biofilms are frequently grown on artificial media conditions that do not mimic physiological environments, leaving the effects of important alternative metabolites largely unexplored. In order to understand the impact of both acidic and acetate-enriched environments in response to fluconazole treatment, we evaluated the whole transcriptome of *C. glabrata* biofilm cells by RNA sequencing. Biofilms were developed for 48 h in RPMI media (with low-glucose concentration, 0.2%), either supplemented or not with 0.5% acetate, and in the presence and absence of fluconazole. This resulted in a total of four different conditions. The total RNA was extracted for 12 independent samples, corresponding to four conditions and three biological replicates. Illumina whole-transcriptome sequencing produced around 1.6 × 10^7^ (±1.4 × 10^6^) reads per sample, which resulted approximately in 98% overall alignment rate. We then performed pair-wise comparisons between conditions to investigate transcriptional responses to fluconazole treatment, either when biofilms were grown in the presence of acetate (RPMI + acetate) or only in glucose (RPMI).

**Fig. 1:**
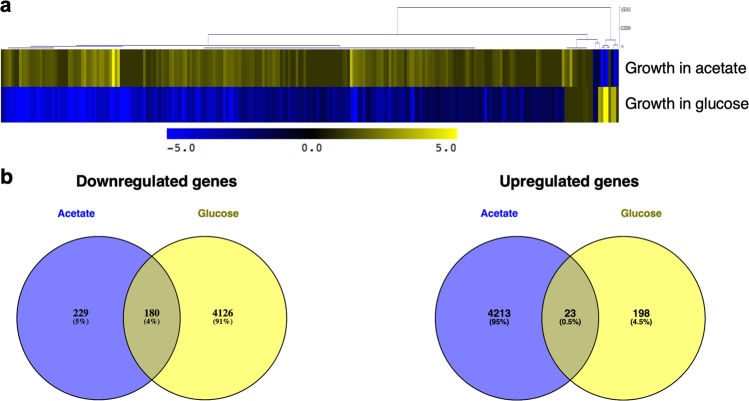
Global transcriptional response of *C. glabrata* biofilm cells to fluconazole treatment when grown in the presence of glucose (RPMI + fluconazole versus RPMI) or in the presence of both glucose and acetate (RPMI + acetate + fluconazole versus RPMI + acetate). **a** Heatmap of all genes differentially expressed (*p* < 0.05) in the presence versus the absence of fluconazole in both growth conditions. **b** Venn diagrams of downregulated (decrease of twofold or lower) and upregulated (greater than twofold) genes in *C. glabrata* biofilm cells due to fluconazole treatment in both growth conditions.

Figure 1a shows a heatmap depicting the gene regulation of *C. glabrata* biofilms in response to both conditions. The adaptation of biofilm cells to the shift from RPMI to RPMI supplemented with acetate, when treated with fluconazole, was accompanied by extensive changes in gene expression. The data show that the transcriptional response of *C. glabrata* biofilm cells to fluconazole is clearly modulated by the presence of acetate (Fig. 1). For both comparisons, we obtained lists of genes significantly up- and downregulated and analyzed those displaying at least twofold regulation (Fig. 1b). We also performed a global functional analysis on the identified genes in order to find significantly enriched GO biological process terms and better understand the biological mechanisms associated with them.

### The transcription of genes involved in DNA replication, ergosterol, and ubiquinone biosynthesis is modulated by the presence of acetate in response to fluconazole treatment

**Fig. 2:**
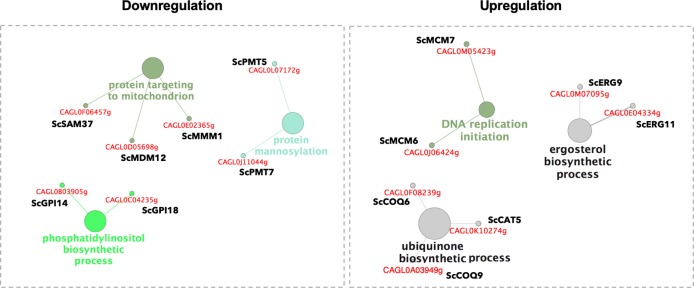
Transcriptional response of *C. glabrata* biofilm cells to fluconazole when grown in the presence of acetate. Network visualization of enriched pathways with the systematic names of *C. glabrata* (downregulated, left panel; and upregulated, right panel) when grown in the presence of acetate and fluconazole was performed by ClueGO analysis. The size of the nodes represents the statistical significance of the terms. The systematic names of *C. glabrata* genes and respective orthologs in *S. cerevisiae* associated with each biological process are shown in red and black, respectively.

**Fig. 3:**
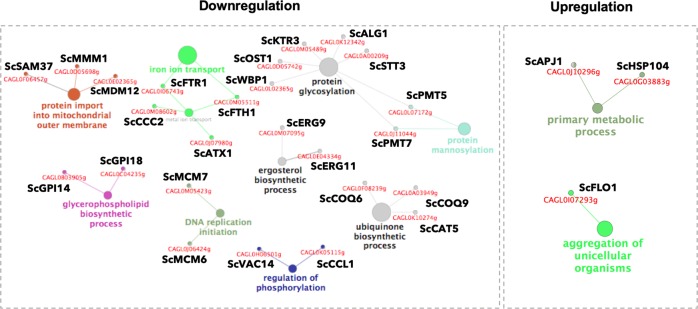
Transcriptional response of *C. glabrata* biofilm cells to fluconazole, grown in the presence of glucose, as sole carbon source. Network visualization of enriched pathways with the systematic names of *C. glabrata* (downregulated, left panel; and upregulated, right panel) when grown in the presence of glucose and fluconazole was performed by ClueGO analysis. The size of the nodes represents the statistical significance of the terms. The systematic names of *C. glabrata* genes and respective orthologs in *S. cerevisiae* associated with each biological process are shown in red and black, respectively.

In order to characterize the transcriptional responses to fluconazole of *C. glabrata* biofilms grown in the presence of acetate, we compared the transcript profiles of samples grown in RPMI media supplemented with acetate and fluconazole, with those grown just in RPMI supplemented with acetate. Upregulated genes were significantly enriched in biological categories such as “DNA replication initiation” (*CgMCM6* and *CgMCM7*), “ergosterol biosynthetic process” (*CgERG9* and *CgERG11*) and “ubiquinone biosynthetic process” (*CgCAT5*, *CgCOQ6*, and *CgCOQ9*; Fig. 2). The overexpression of both Mcm6p and Mcm7p, which are associated with the MCM complex that initiates and regulates DNA replication,^17^ may be indicative of increased cell proliferation that ultimately may lead to chromosomal instability. This hypothesis is in agreement with previous observations correlating chromosomal abnormalities in several *Candida spp*. with exposure to fluconazole.^18^ However, this response has never been associated with the presence of acetate or other non-fermentable carbon sources during *C. glabrata* biofilm growth. Considering the overexpression of ergosterol biosynthetic genes, this is one of the most common mechanisms of resistance in response to fluconazole treatment in *Candida spp*.^18^ The mechanism of action of fluconazole involves the inhibition of cytochrome P450 enzyme lanosterol demethylase, encoded by *ERG11*.^19^ This reaction occurs due to the binding of a free nitrogen atom of the azole ring to an iron atom within the heme group of the enzyme. This binding prevents oxygen activation and consequently demethylation of lanosterol, resulting in the inhibition of ergosterol biosynthesis. Moreover, Erg9p is also involved in ergosterol biosynthesis but functions upstream of lanosterol demethylase in the biosynthetic pathway.^20^ As ergosterol is an important component of fungal cell membranes, any impairment in the ergosterol biosynthetic pathway results in increased cellular permeability leading to the disruption of cell membranes. Thus, ergosterol overexpression confers protection against fluconazole treatment by maintaining sterol homeostasis. Lastly, the three genes encoding proteins involved in the ubiquinone biosynthesis, Coq6p, Cat5p and Coq9p, are part of a multi-subunit complex of nine proteins required for ubiquinone biosynthesis.^21–24^ Ubiquinone, also known as Coenzyme Q, is an essential component of the electron transport chain, carrying electrons from Complexes I and II to Complex III in mitochondria. These proteins are required for respiratory growth and gluconeogenic gene activation.^21–23^ Indeed, during growth on non-fermentable carbon sources, such as acetate, *Candida* cells induce gluconeogenesis in order to obtain sugar phosphates for the synthesis of essential cellular components.^25^ Collectively, these data suggest that fluconazole increases chromosome instability, ergosterol biosynthesis and endogenous respiration in *C. glabrata* biofilm cells grown in the presence of acetate (Fig. 2). When grown in the presence of glucose as sole carbon source, all of these genes were found to be downregulated (Fig. 3), suggesting that these pathways are regulated in a carbon source-dependent manner.

### Reduction of mitochondrial activity appears to be induced in response to fluconazole treatment in a manner independent of carbon source

On the other hand, downregulated genes were significantly enriched in biological processes related to alterations in mitochondrial biogenesis and cell wall organization, as a response to fluconazole treatment. Genes involved in “protein targeting to mitochondrion” (*CgSAM37*, *CgMDM12*, and *CgMMM1*) codify a set of translocases that are part of a Sorting Assembly Machinery (SAM) complex that mediates the insertion of beta-barrel proteins into the mitochondrial outer membrane.^26–28^ The core of this complex is composed of Sam50p, Sam37p and Sam35p,^29–32^ but other proteins such as Mdm12p and Mmm1p are required to successfully complete this process.^33^ This transcriptional response is in accordance with previous transcriptomics and metabolomics analysis in *C. albicans* that suggest a downregulation of mitochondrial activity as a result of biofilm maturation.^34–37^ Other studies have also suggested that the reduction of mitochondrial activity might be beneficial for biofilm growth, conferring protection against oxidative damage and promoting prolonged survival of biofilm cells.^38,39^ The downregulation of these genes provides additional evidence that the loss of mitochondrial functions is associated with fluconazole resistance in *C. glabrata*. Other biological categories also found to be downregulated due to fluconazole treatment include “protein mannosylation” (*CgPMT5* and *CgPMT7*) and “phosphatidylinositol biosynthetic process” (*CgGPI14* and *CgGPI18*; Fig. 2). The cell wall glycoproteins of *Candida spp*. are modified by *N-* and *O-*linked glycosylation and are attached to the polysaccharides at the cell wall by either covalent links or glycosylphosphatidylinositol (GPI) anchors.^40^ The protein O-mannosyltransferases (PMTs) are an evolutionarily conserved, essential family of proteins that mannosylate distinct targets in the endoplasmic reticulum.^41^ In *C. albicans*, where these proteins have been well studied, this posttranslational modification was shown to be involved in morphogenesis, adhesion and antifungal resistance, suggesting that PMTs can be studied as potential antifungal targets.^42–44^ Both Gpi14p and Gpi18p are also mannosyltransferases but specifically responsible for adding α-1,4-linked and α-1,6-linked mannoses to GPI, respectively.^45–47^ As in fungi all of these genes have several subfamily members that are highly redundant, the effect of their downregulation is unclear. Interestingly, our data show that upon fluconazole treatment, biofilm cells grown only in the presence of glucose exhibit the same transcriptional pattern of regulation for all categories mentioned above (Figs 2 and 3). We conclude that these transcriptional responses to fluconazole are independent of the carbon source.

### In glucose-grown biofilm cells, fluconazole treatment induces the overexpression of genes involved in metabolism, protein folding, and adhesion

To characterize the transcriptional response of glucose-grown biofilm cells to fluconazole, we compared the transcript profile of samples grown in RPMI and fluconazole with those grown in RPMI. Upregulated genes due to fluconazole treatment were significantly enriched in processes related to “primary metabolic process” (*CgAPJ1* and *CgHSP104*) and aggregation (*CgFLO1*, also known as *CgEPA1*) (Fig. 3). Apj1p is a J protein cochaperone of the HSP40 family while Hsp104p is a general anti-stress chaperone that in conjunction with other proteins helps to disassemble protein aggregates accumulated due to stress.^48,49^ Heat-shock proteins (HSPs) are induced by several types of stress and have broadly protective functions.^50^ Their expression has been associated with biofilm formation and drug resistance in *Candida spp*., reinforcing their potential as antifungal targets.^48,50,51^ Some links between azole resistance and protein folding functions have also been reported in *C. albicans*.^52,53^ *CgEPA1* is a GPI-anchored cell wall protein that participates in cell-cell interactions, allowing adherence to host epithelial tissues.^54^ In *C. glabrata*, Epa1p is the major adhesin mediating adhesion and its overexpression is associated with resistance to fluconazole.^55^

### Iron acquisition and glycosylation appear to be attenuated by fluconazole treatment when glucose is present, as sole carbon source

The transcriptional responses to fluconazole when *C. glabrata* biofilm cells were grown in the presence of glucose are associated with a higher number of differentially regulated genes when compared with the acetate-dependent responses described above. Downregulated genes were significantly enriched in several processes (Fig. 3), including “iron ion transport” (*CgFTR1*, *CgFTH1*, *CgCCC2* and *CgATX1*). Since Ftr1p, Fth1p and Ccc2p are part of the high-affinity iron uptake system in *C. glabrata*, and Atx1p is also involved in iron absorption, downregulation of these genes may result in perturbed iron homeostasis.^56^ Given that iron acts as a cofactor for numerous metalloproteins involved in several fundamental cellular processes, such as oxygen transport, respiration, energy metabolism and DNA synthesis and repair, perturbations in iron homeostasis also have a direct impact on these processes. This is consistent with some of the downregulated categories found under the same conditions, namely, ergosterol biosynthesis. Additionally, genes involved in “protein glycosylation” (*CgWBP1*, *CgOST1*, *CgKTR3*, *CgALG1* and *CgSTT3*) were also found to be downregulated in response to fluconazole (Fig. 2). The cell wall glycoproteins in *Candida spp*. can be *N-* or *O-*glycosylated. The *N-*linked glycosylation pathway requires the action of different glycosyltransferases and comprises two sequential stages.^40,57^ The first stage involves the synthesis of a dolichol-linked glycan precursor and its transfer to a nascent protein in the rough endoplasmic reticulum. Alg1p is involved in the synthesis process and Wbp1p, Stt3p, and Ost1p are transmembrane subunits of the oligosaccharyl transferase complex (OST) that mediate the transference.^57^ The second stage includes the *N-*linked glycan processing and maturation in the rough endoplasmic reticulum and Golgi. *CgKTR3*, a member of the *KRE2/MNT1* gene family, may be involved in this last stage but also in *O-*linked protein glycosylation.^58^ A reduction in the levels of glycosylation will impact the composition of the cell wall and thus the interaction between the biofilm cells. This is consistent with data from *C. albicans* showing that changes in carbon source affect cell wall architecture, the cell wall proteome and cell wall organization.^59–61^ Besides glycosylation, some proteins involved in the “regulation of phosphorylation” (*CgCCL1* and *CgVAC14*) also appear to be downregulated in response to fluconazole treatment (Fig. 3). *CgCCL1* positively regulates transcription by Polymerase II by stimulating phosphorylation of RNA polymerase II C-terminal domain, with mutants showing increased competitive fitness in *Saccharomyces cerevisiae*.^62^ Vac14p controls the synthesis of phosphatidylinositol-3,5-biphosphates, impacting several mechanisms such as organelle morphology, membrane recycling, and ion transport.^63^ Altogether, these biological categories represent a deregulation in mitochondrion function, cell cycle, cell wall composition, iron transport, and post-translation modifications, such as mannosylation, glycosylation, and phosphorylation.

### Validation of RNA-seq results with quantitative real-time PCR (qRT-PCR)

**Fig. 4:**
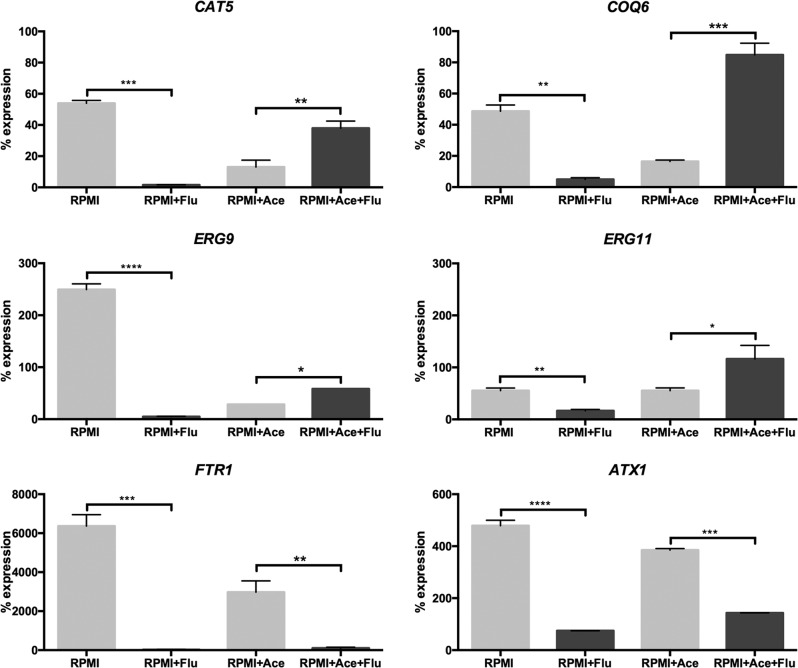
Validation by qRT-PCR of genes that were differentially expressed (*CAT5*, *COQ6*, *ERG9*, *ERG11*, *FTR1*, and *ATX1*) in response to fluconazole in *C. glabrata* biofilm cells grown with glucose as sole carbon source (RPMI; pH 7.0) or supplemented with acetate (RPMI + Ace; pH 5.0). Graphs show percentage expression of each gene compared to a housekeeping gene, *PGK1*. The error bars show standard deviation. *p < 0.05, **p < 0.01, ***p < 0.001, ****p < 0.0001 were considered statistically significant relative to untreated *C. glabrata* biofilm cells.

In order to validate the RNA-seq findings, qRT-PCR was performed on all conditions for six genes that are key members of the most significant differentially expressed categories found by GO analysis: *CAT5*, *COQ6*, *ERG9*, *ERG11*, *FTR1*, and *ATX1* (Fig. 4). All of these genes were also tested when *C. glabrata* biofilm cells were grown in the presence of fluconazole, and when using glucose as sole carbon source at pH 5.0, to check whether the pH influenced the changes in gene expression (Supplementary Fig. 1). Significantly, the changes in expression observed by RNA-seq were confirmed by qRT-PCR for all the tested genes. Furthermore, the changes in expression observed between growth conditions were independent of the pH (Fig. 4 and Supplementary Fig. 1).

### CgMge1 drives fluconazole resistance in acidic and acetate-enriched environments

**Fig. 5:**
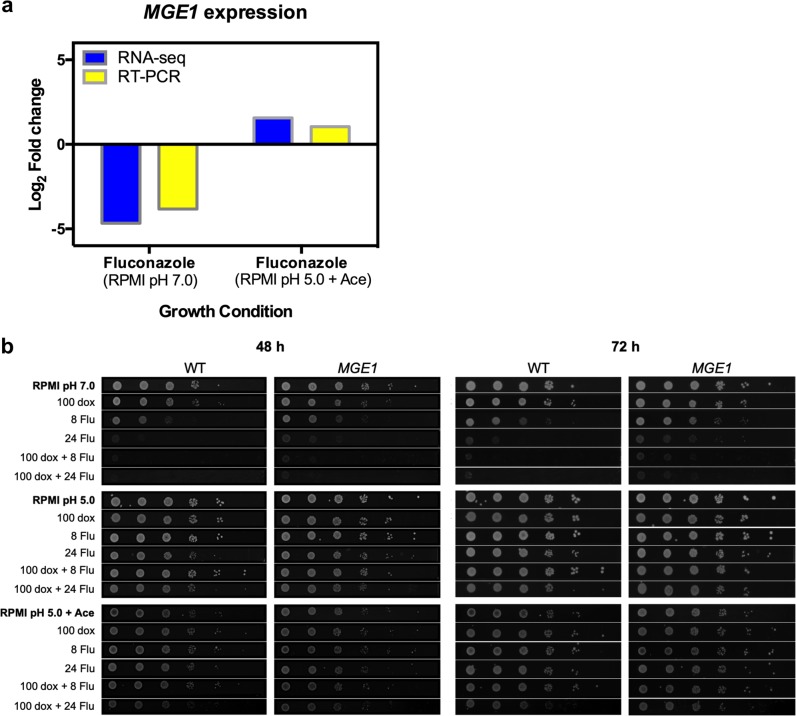
Fluconazole susceptibility is modulated by pH, carbon source and *MGE1* expression in *C. glabrata*. **a** Log_2_ fold-change comparison of RNA-seq (blue bars) and quantitative RT-PCR (yellow bars) for *MGE1* expression in response to fluconazole treatment when *C. glabrata* cells are growing either using glucose as sole carbon source (RPMI pH 7.0) or in the presence of acetate (RPMI pH 5.0 + Ace). **b** Serial dilutions of *wild-type* strain (WT; 2001HTL strain transformed with the empty plasmid as a control) and overexpression strain (*MGE1*; 2001HTL strain transformed with a plasmid expressing *CgMGE1* ORF, together with its terminator, from the *CgTDH3* promoter^65^) were spotted on RPMI medium containing 0.2% glucose at pH 7.0 or pH 5.0, and/or containing acetate (ace; 0.5% v/v), and/or fluconazole (flu; 8 or 24 µg/mL) and/or doxycycline (dox; 100 µg/mL). Pictures were taken after 48 h and 72 h of incubation at 37 °C.

*C. glabrata* is able to colonize and infect acidic and acetate-enriched anatomical sites, including the gastrointestinal tract and vagina. We identified a set of genes that are differently regulated due to fluconazole treatment, when cells are growing in conditions mimicking those acidic environments. Considering the overexpression of both *CgERG9* and *CgERG11* in response to fluconazole, when *Candida* biofilm cells are growing in the presence of acetate (Fig. 2), we postulated that *C. glabrata* would display increased resistance to fluconazole under those conditions. To physiologically validate our transcriptional data, *C. glabrata* cells were grown in the presence of fluconazole and/or doxycycline, an iron chelator reported to have a synergistic effect with fluconazole.^64–66^ As a control strain, we used an overexpression mutant for *MGE1* driven by the *CgTDH3* promoter in the 2001HTL strain (Fig. 5).^65^ This gene encodes a putative mitochondrial matrix cochaperone, which is a known suppressor of fluconazole susceptibility in both *C. albicans* and *C. glabrata*.^65^ Since *CgMGE1* is upregulated when cells are treated with fluconazole, under acidic environments (Fig. 5a), we hypothesized that WT cells would display the same phenotype as *MGE1*-overexpressing cells, when grown under similar conditions. We tested this, and all experiments were performed in triplicate, showing consistent results among all the independent assays. Growth conditions were maintained by supplementing RPMI medium containing 0.2% glucose with the respective stressor and adjusting the pH to 7.0 or 5.0. This enabled us to compare our physiological data with the transcriptional outputs.

Phenotypes from control conditions (RPMI pH 7.0) for the *MGE1*-overexpressing strain were in agreement with previously published data, where *CgMGE1* overexpression was shown to play a role in regulating susceptibility to fluconazole (Fig. 5b).^65^ As expected, cells grown under acidic conditions, such as RPMI at pH 5.0 and RPMI at pH 5.0 supplemented with 0.5% acetate, were more resistant to fluconazole and doxycycline than those grown only in RPMI at pH 7.0 (Fig. 5b). Interestingly, the synergistic effect of doxycycline was strongly modulated by pH and the carbon source, since this effect was abolished under acidic pH, and moderately restored for the highest concentration of fluconazole in the presence of acetate (Fig. 5b). As we predicted, both WT and *MGE1*-overexpressing cells displayed the same phenotype under acidic conditions (Fig. 5b). We can conclude from these data that overexpression of *MGE1* under acidic conditions (confirmed both by RNA-seq and RT-PCR; Fig. 5a) causes a decrease in the susceptibility of *C. glabrata* to fluconazole (Fig. 5b). Our data also highlight the importance of mimicking distinct environmental niches as local inputs significantly affect the responses of *Candida* cells to antifungal treatment.

### Overexpression of CgMGE1 reduces toxic sterol formation

**Fig. 6:**
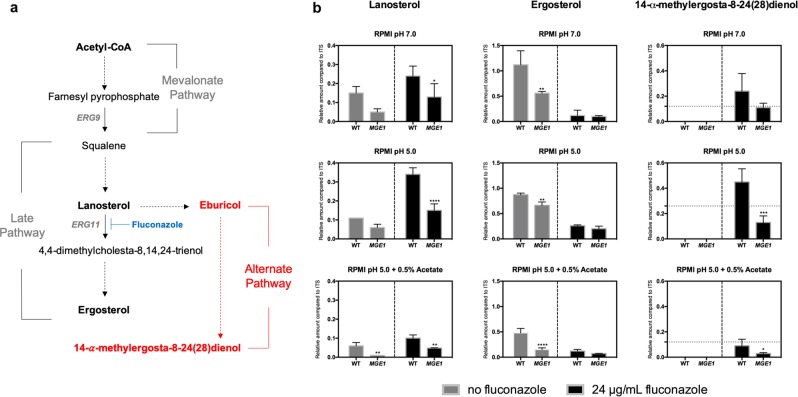
*MGE1* overexpression modulates the aberrant flux of sterols following fluconazole treatment. **a** Schematic representation of sterol biosynthetic pathways. Erg11 is the primary target of fluconazole. Red names represent toxic fungistatic sterols. **b** *CgMGE1* overexpression reduces toxic sterol formation. Cells were grown in RPMI medium containing 0.2% glucose at pH 7.0, or pH 5.0, and/or containing 0.5% acetate, in the presence or absence of fluconazole for 24 h. Sterol levels were determined by GC-MS and are displayed for lanosterol, ergosterol, and 14-methylergosta-8,24(28)-dien-3β,6α-diol. The values were calculated relative to the internal standard (ITS; cholestane). The error bars show standard error of the mean. *p < 0.05, **p < 0.01, ***p < 0.001, ****p < 0.0001 were considered statistically significant relative to *C. glabrata* WT cells. Gray dotted grid lines in each condition represent the mean obtained for ergosterol content for the same *MGE1*-overexpressing cells.

**Fig. 7:**
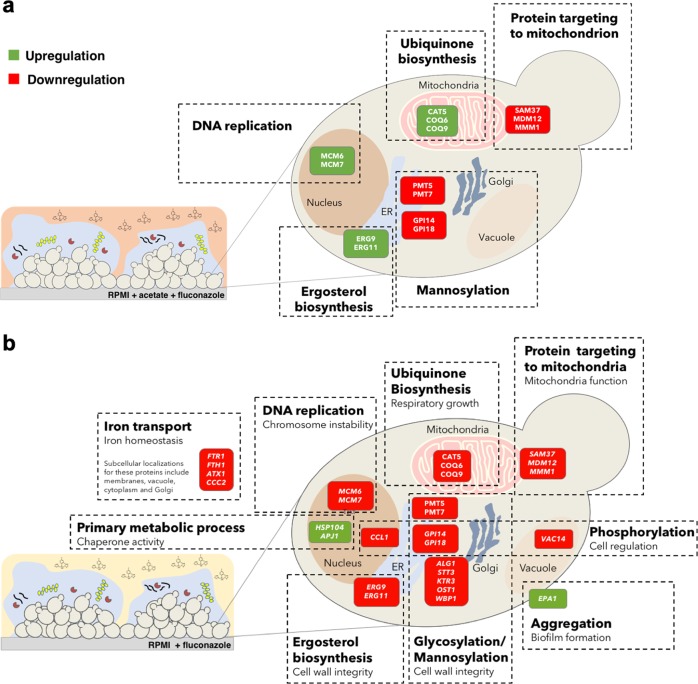
Schematic overview of the transcriptional responses of *C. glabrata* biofilms to fluconazole. Differentially expressed genes upon fluconazole treatment when cells are grown in the presence of **a** glucose and acetate or **b** glucose.

Ergosterol biosynthesis is inhibited in the presence of fluconazole, favouring the formation of toxic sterols due to the accumulation of lanosterol (Fig. 7a). Previous work has demonstrated that the overexpression of *CgMGE1* reduces the metabolic flux to toxic sterol formation.^65^ Therefore, we performed gas chromatography-mass spectrometry (GC-MS) analysis to investigate the possible role of sterols in mediating fluconazole resistance, which is higher when *C. glabrata* cells grow in acidic environments and when *MGE1* is upregulated. Sterols were isolated from both WT and *MGE1*-overexpressing cells grown in RPMI at pH 7.0, RPMI at pH 5.0 and RPMI at pH 5.0 + 0.5% acetate, in the presence and absence of fluconazole (Fig. 6b). In addition to sterols such as ergosterol and lanosterol, we detected other sterols that were less abundant or could not be identified (Supplementary Fig. 2). This approach was validated by using an alternative sterol quantitation method (SQM; Supplementary Fig. 3).^67^

As can be seen from Fig. 6b, fluconazole treatment leads to lanosterol accumulation and ergosterol depletion under all the growth conditions examined. This accumulation favors the formation of toxic sterols such as 14-methylergosta-8,24(28)-dien-3β,6α-diol. Remarkably, *MGE1*-overexpressing cells displayed reduced levels of this toxic sterol (Fig. 6b). This reduction is statistically significant only for cells grown under acidic conditions (*p* < 0.001 and *p* < 0.05 for RPMI pH 5.0 and RPMI pH 5.0 + 0.5% acetate, respectively). Additionally, the balance between ergosterol and 14-methylergosta-8,24(28)-dien-3β,6α-diol in these cells is higher for RPMI at pH 5.0 and RPMI at pH 5.0 + 0.5% acetate (Fig. 6b). By reducing the metabolic flux towards 14-methylergosta-8,24(28)-dien-3β,6α-diol, other sterol intermediates can migrate to the plasma membrane and replace the essential ergosterol functions. Considering that under these conditions, *C. glabrata* cells present higher fluconazole resistance (Fig. 5b), these data present additional evidence for the role of *MGE1* in reducing the levels of toxic sterols and, consequently, decreasing susceptibility to fluconazole.

## Discussion

Natural niches of *C. glabrata* include the human gastrointestinal and vaginal tracts,^68^ environments particularly rich in alternative carbon sources and often limited in the amount of glucose.^11^ In order to thrive in these changing nutrient environments, *C. glabrata* has evolved sophisticated regulatory mechanisms, including major metabolic changes that have been associated with virulence and, in particular, biofilm formation.^15,16,69^ Once established, *Candida* biofilms are very difficult to eradicate due to their intrinsic tolerance to antifungals. In fact, azole resistance among *Candida* species, especially *C. glabrata*, is currently one of the greatest clinical challenges.^2,4,7^ Here, we investigated the effect of different carbon sources on fluconazole-treated *C. glabrata* biofilms by using a RNA-seq approach. For that, biofilms were grown in the presence of low glucose or low glucose plus acetate, a non-fermentable carbon source frequently found in several host niches.^14,70^ The differentially regulated genes identified in both growth conditions allowed us to decipher the potential molecular mechanisms associated with carbon adaptation and fluconazole resistance. Indeed, since most of the genes are still uncharacterized in *C. glabrata*, their function was predicted based on their orthologs in the phylogenetically closely related yeast *S. cerevisiae*.

Depending on the growth condition, some genes were found to be differently regulated in response to fluconazole. These included genes encoding proteins involved in DNA replication, mitochondrial function, and ergosterol biosynthesis (Figs 2 and 3). While this observation could be anticipated for the genes involved in DNA replication and mitochondrial function, as metabolism is finely coordinated with cell growth, chromosome replication and cell division, it was quite surprising to find that the carbon source affects the degree to which fluconazole induces ergosterol-related genes. As sterol biosynthesis requires heme, we hypothesize that the expression of these genes is also dependent on the status of iron acquisition in the cells. Indeed, the expression profile of iron-related genes is finely tuned with those involved in the expression of ergosterol (Figs 2–4). Curiously, even with all of these impairments, *C. glabrata* cells from biofilms still survive under high concentrations of fluconazole.^16^ In fact, it was already shown that deprivation of iron does not seem to affect the ability of cells to form biofilms in *C. albicans*.^71^ Moreover, the lack of iron enhances membrane fluidity and drug diffusion in *C. glabrata* planktonic cells,^66^ consistent with the impairment in ergosterol biosynthesis. On the other hand, our analyses provided additional evidence that the loss of mitochondrial function is associated with fluconazole resistance, independently of the growth condition. Additionally, while physiologically validating our transcriptional outputs, we realized that *C. glabrata* Mge1, a cochaperone involved in iron metabolism and protein import into the mitochondria, might influence fluconazole susceptibility during carbon and pH adaptation. We confirmed this experimentally (Fig. 6), thereby reinforcing the role of *CgMGE1* in mediating fluconazole resistance by reducing toxic sterol formation.^65^

In this study, we did not observe statistically significant deregulations regarding several well-known genes involved in fluconazole resistance in planktonic *C. glabrata* cells. These include the induction of efflux pumps, encoded by the *ABC*-transporter genes, such as *CgCDR1*, *CgCDR2* (also known as *CgPDH1*), and Cg*SNQ2*, which are regulated mainly by the transcription factor *CgPDR1*.^2^ Our data suggest that their expression levels differ markedly between biofilms and planktonic cells (Supplementary Fig. 4). This observation is consistent with previous work done in *C. albicans*.^72^ Mutants knocked out in these genes were hypersusceptible to fluconazole when growing as planktonic cells, but not when growing as biofilms.^72^ This may reflect altered responses due to the characteristics intrinsically related with the biofilm lifestyle, such as growth on a surface, the presence of a protective extracellular matrix, differences in nutrient requirements and metabolism, and/or quorum-sensing mechanisms.^73^

Based on the data presented here, we propose a model in which resistance to fluconazole in *C. glabrata* biofilms is modulated by the available carbon source and pH (Fig. 7). This is particularly relevant for a set of genes involved in DNA replication, ergosterol, and ubiquinone biosynthesis. When grown in the presence of glucose, responses to fluconazole treatment are extensive and involve the deregulation of several cellular functions (Fig. 7a). On the other hand, when acetate is present in the growth media, fluconazole treatment impacts mainly chromosome instability, cell wall integrity, and mitochondrial function (Fig. 7b).

Our analyses provide a global overview of the genetic and regulatory mechanisms developed by this microorganism in response to antifungal treatment, and highlights the impact of acidic environments on these processes. These alterations at transcriptional level following fluconazole treatment, either in the presence of glucose or acetate, enhance the robustness of *C. glabrata* biofilms to tolerate this antifungal. Our data reveal new roles of uncharacterized *C. glabrata* genes and provide more knowledge towards the identification of new potential antifungal targets. Understanding the mechanisms behind fluconazole resistance may improve and facilitate the development of new antifungal compounds to treat *Candida* infections.

## Methods

### Media, strains, and growth conditions

Biofilm experiments were done using *Candida glabrata* ATCC 2001. This strain was subcultured on Sabouraud dextrose agar (SDA, Difco, Le Pont de Claix, France) medium at 37 °C for 24 h and then inoculated in Sabouraud dextrose broth (SDB; Difco) medium at 37 °C for 20–24 h while shaking at 120 rpm. Yeast cells were harvested by centrifugation after the incubation period (6500 × *g*, for 10 min at 4 °C), washed twice in phosphate buffered saline (PBS; pH 7.0, 0.1 M), and the cellular concentration was adjusted to 1 × 10^7^ cells/mL using an improved Neubauer chamber. All experimental assays were carried out in filter-sterilized RPMI 1640 medium with l-glutamine (Sigma, R6504) and buffered with 0.165 M morpholinepropanesulfonic acid at pH 7.0 or 5.0. Fluconazole (Sigma; F8929), doxycycline (Sigma, D9891) and acetic acid (Sigma, A6283) were added to the medium at concentrations of 8 or 24 µg/mL, 100 µg/mL, and 0.5% (v/v), respectively (unless otherwise stated). Depending on the assay, autoclave-sterilized and precooled agar was added to the medium. Cell cultures containing fluconazole or doxycycline were always kept in the dark. Detailed information about *MGE1*-overexpressing strain and respective wild-type control can be found in Demuyser et al.^65^

### Biofilm development

*Candida* biofilms were developed on 24-well polystyrene plates (Orange Scientific, Braine-l’Alleud, Belgium; 12 wells for each condition) containing 1 mL of *C. glabrata* cell suspension (1 × 10^7^ cells/mL in RPMI 1640) per well. The plates were then incubated at 37 °C under agitation at 120 rpm. After 24 h, 500 μL of media in each well was removed and an equal volume of fresh media was added according to each condition (RPMI at pH 5.0 supplemented with 0.5 % (v/v) acetic acid; RPMI at pH 5.0 supplemented with 0.5% (v/v) acetic acid and 1250 µg/mL fluconazole; RPMI at pH 7.0; and RPMI at pH 7.0 supplemented with 1250 µg/mL fluconazole). The concentration of fluconazole was selected according to previous studies with *C. glabrata biofilms*.^16^ Plates were then incubated for additional 24 h at 37 °C under agitation at 120 rpm.

### RNA isolation

Following *Candida* biofilm development, each well was gently washed with 1 mL of PBS to remove non-adherent cells. Then biofilms were scraped from the plates with 500 µL of PBS per well, sonicated for 30 s at 30% amplitude (Ultrasonic Processor, Cole-Parmer, IL, USA) in order to separate cells from matrix^74^ and then harvested by centrifugation at 8000 × *g* for 10 min at 4 °C. Cells drops were flash frozen in liquid nitrogen and total RNA was isolated from frozen cell pellets using the RiboPure Yeast kit (Ambion, Life Technologies, USA), according to manufacturer’s instructions. All samples were treated with Turbo DNAse (Ambion) to remove residual DNA, according to manufacturer’s instructions. Total RNA yield was quantified using the Nanodrop 1000 (Thermo Scientific) and RNA quality (RNA Integrity Number (RIN) values ≥ 7.0) assessed on a Bioanalyzer 2100 (Agilent Technologies).

### RNA sequencing

Library preparation and RNA sequencing were performed at Edinburgh Genomics (Scotland, UK). All samples were prepared in biological triplicates and subject to removal of ribosomal RNA before complementary DNA (cDNA) library generation. From these libraries, 50 base-paired-end sequence reads were produced with Illumina Hiseq 2000. The raw sequence data in fastq format as well as the processed data have been deposited in NCBI’s Gene Expression Omnibus^75^ and are accessible through GEO Series accession number GSE121074.

### Data analysis

Raw fastq files were successively processed in the following order through Fastqc (v. 10.1), Trimgalore (v. 3.1), Samtools (v. 1.19), Bowtie2 (v. 2.1), and Htseq (v. 5.4). Genome alignment was conducted against the C_glabrata_CBS138_version_s02-m04-r05_chromosomes.fasta file provided by *Candida* Genome Database.^76^ Aligned data were quality-controlled via the Partek Genomics Suite software (v. 6.6), according to the manufacturer’s instructions. Gene expression analysis was performed using Partek Genomics Suite software using a log_2_ data transformation as the Partek recommended default. Gene ontology (GO) term analysis was performed in parallel through the *Candida* Genome Database GO Term Finder and the Cytoscape (v. 3) Clue GO plugin.^77^ Network construction was performed with Cytoscape V.3 freeware,^78^ venn diagrams through Venny online freeware (v. 2.0.2) and heatmaps with TM4 MultiExperiment Viewer (MeV, v. 4.9). A statistical comparison among GO term enrichment percentages was performed with GraphPad Prism (v. 6) using the Student’s *t*-test for two-tailed data. Data represent three independent biological replicates for each condition.

### Quantitative real-time PCR (qPCR)

Real-Time quantitative PCR of cDNA samples was carried out in a CF X96 Real-Time PCR System from Bio-Rad Laboratories using Power SYBR® Green PCR Master Mix (Applied Biosystems, CA, USA), according to manufacturer’s instructions. The High-Capacity cDNA Reverse Transcription Kit (Invitrogen, CA, USA) was used to synthesize the cDNA in a 20 µL reaction containing 1 µg of total RNA, according to the manufacturer’s instructions. The primers used to amplify the selected genes and the thermocycling conditions are described in Supplementary Table 1. The reaction mixture was set up in a total volume of 20 μL using 10 μL of SYBR Green PCR Master Mix, 0.3 μM of each primer and 4 μL of each synthesized cDNA sample (diluted 1:20) and nuclease-free water. A negative control without template was conducted for each gene in each PCR run, and a control for DNA contamination was implemented by using the purified RNA samples as templates. The housekeeping gene, *PGK1*^79^ was used to normalize the gene expression. Experiments were performed in triplicate for three independent biological samples. Statistical analysis were performed using two-way ANOVA with Bonferroni correction.

### Sterol measurement

Sterols were extracted according to the method described in Morio et al.^80^ with a few adaptations. In summary, cells were grown for 24 h in RPMI medium, with or without 24 μg/mL fluconazole. The cells were collected, resuspended in saponification medium, and subjected to vortex mixing. The samples were incubated for 1 h at 80 °C, after which 1 mL of water and 4 mL of hexane were added. After mixing, the two layers were allowed to separate. For spectrophotometrical analysis, UV-transmittable 96-well microtiter plates (Costar Corning) were used to allow measurement of the OD_281_ and OD_230_. A formula from Arthington-Skaggs et al.^67^ was used to measure the percentages of ergosterol (corrected for cellular wet weight and resuspension volume). For GC-MS analysis, the sterols were extracted twice with hexane, which was then evaporated by vacuum centrifugation. The sterols were resuspended in 100 μL silylating mixture (Sigma) and incubated at room temperature for 30 min. Finally, a corrected cellular wet weight resuspension volume of hexane was added and the samples were immediately stored at −20 °C for later analysis by GC-MS. One microliter of the sample was injected into a gas chromatograph-mass spectrometer (Shimadzu QP2010 Ultra Plus) equipped with an HP-5ms nonpolar column (Agilent) (30 m in length, 0.25-mm inner diameter [id.]; 0.25-µm thin layer). Helium was used as carrier gas with a flow rate of 1.4 mL/min. Injection was carried out at 250 °C in split mode after 1 min and with a ratio of 1:10. The temperature was first held at 50 °C for 1 min and then allowed to rise to 260 °C at a rate of 50 °C/min, followed by a second ramp of 2 °C/min until 325 °C was reached; that temperature was maintained for 3 min. The mass detector was operated in scan mode (50 to 600 atomic mass units [amu]), using electron impact ionization (70 eV). The temperatures of the interface and detector were 290 and 250 °C, respectively. A mix of linear *n*-alkanes (from C_8_ to C_40_) was injected to serve as external retention index markers. Sterols were identified by their retention time relative to the internal standard (cholestane) and specific mass spectrometric patterns using AMDIS version 2.71. The deconvoluted spectra were matched to GC-MS libraries described in Müller et al.^81^ and NIST/EPA/NIH version 2011. Analysis was performed by integration over the base ion of each sterol, and abundance was calculated relative to the internal standard, comparing the relative peak areas of the compounds across treatments using two-way ANOVA with Bonferroni correction.

### Reporting summary

Further information on research design is available in the Nature Research Reporting Summary linked to this article.

## Supplementary information

Supplementary Material

Reporting Summary Checklist

## Data Availability

The datasets generated in this study are available at public repositories or from the corresponding author upon request. The raw RNA-seq data in fastq format, as well as the processed data have been deposited in NCBI’s Gene Expression Omnibus and are accessible through GEO Series accession number GSE121074.
