# Supplementary material for: Transcriptional responses of Candida glabrata biofilm cells to fluconazole are modulated by the carbon source
Source: NPJ Biofilms Microbiomes. 2020 Jan 23;6:4. doi: 10.1038/s41522-020-0114-5 (PMC6978337; doi:10.1038/s41522-020-0114-5)
Supplement: Supplementary file 1 — Supplementary Material [file 41522_2020_114_MOESM1_ESM.pdf]

Supplementary Material

Transcriptional responses of *Candida glabrata* biofilm cells to fluconazole are modulated by the carbon source

Rosana Alves[1], Stavroula L Kastora[2], Alexandra Gonçalves[1], Nuno Azevedo[3], Célia Rodrigues[3], Sónia Silva[3], Liesbeth Demuyser[4,5], Patrick Van Dijck[4,5], Margarida Casal[1], Alistair J P Brown[2,6], Mariana Henriques[3] and Sandra Paiva[1*]

[1] Centre of Molecular and Environmental Biology, Department of Biology, University of Minho, Braga, Portugal

[2] Aberdeen Fungal Group, University of Aberdeen, Institute of Medical Sciences, Foresterhill, Aberdeen, United Kingdom

[3] Centre for Biological Engineering, University of Minho, Braga, Portugal

[4] VIB-KU Leuven Center for Microbiology, Flanders, Belgium

[5] Laboratory of Molecular Cell Biology, Institute of Botany and Microbiology, KU Leuven, Leuven, Belgium

[6] MRC Centre for Medical Mycology, University of Exeter, Geoffrey Pope Building, Stocker Road, Exeter, United Kingdom

*** Correspondence:** Dr. Sandra Paiva, Centre of Molecular and Environmental Biology, Department of Biology, University of Minho, Campus de Gualtar, 4710-057 Braga, Portugal. spaiva@bio.uminho.pt

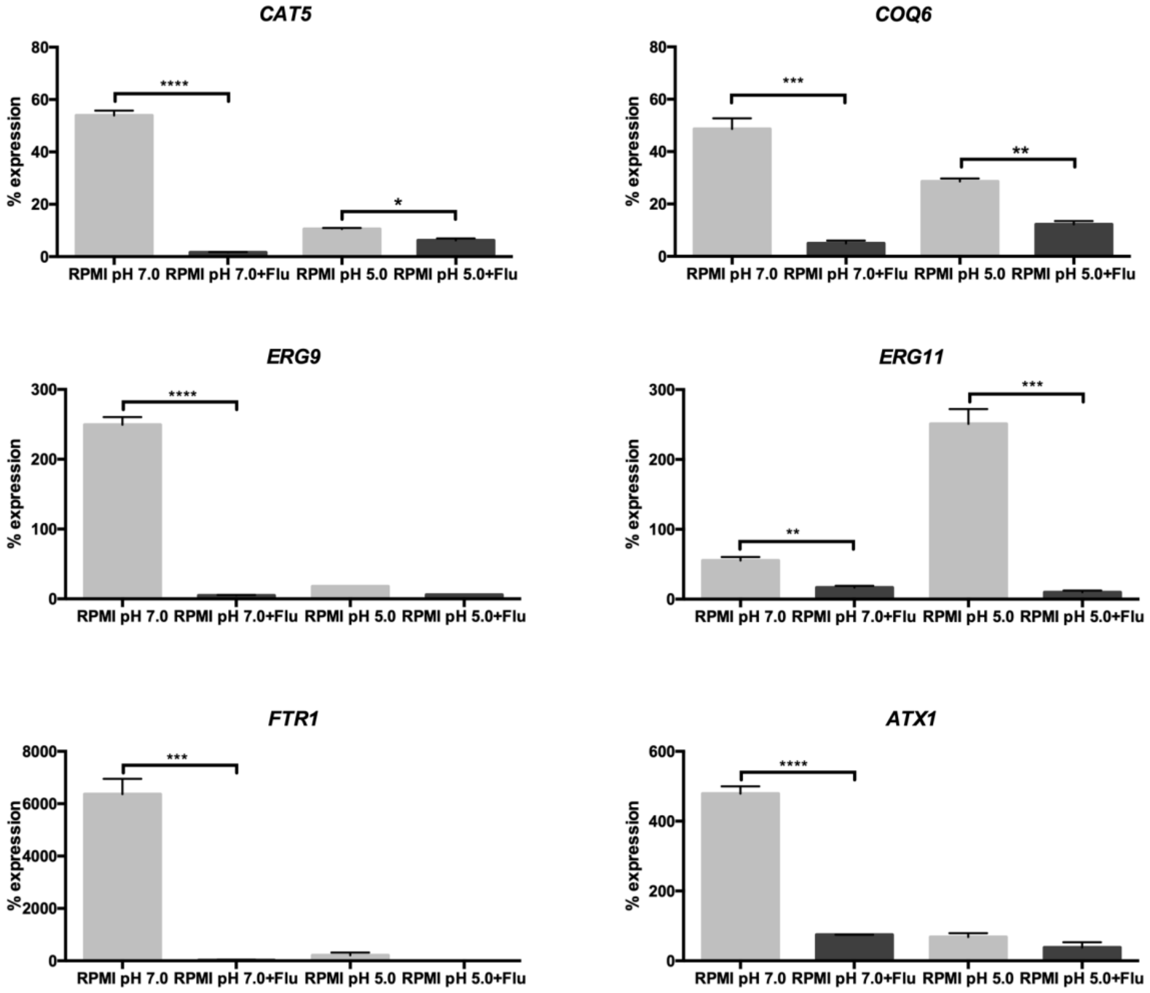

Supplementary Figure 1. Expression of *CAT5*, *COQ6*, *ERG9*, *ERG11*, *FTR1* and *ATX1* assessed by qRT-PCR in response to fluconazole in *C. glabrata* biofilm cells grown using glucose as sole carbon source (RPMI pH 7.0 vs RPMI pH 5.0). Graphs show percentage expression of each gene compared to a housekeeping gene, *PGK1*. Error bars show standard deviation. * $p < 0.05$, ** $p < 0.01$, *** $p < 0.001$, **** $p < 0.0001$ were considered statically significant relative to untreated *C. glabrata* biofilm cells.

IUPAC name	Trivial name	RT	RRT	Molecular ion TMS-derivative (g/mol)	Ion used for quantification (m/z)	Relative abundance RPMI pH 7.0 WT Mean	SEM	WT + Fluconazole Mean	SEM	*MGE1* Mean	SEM	*MGE1* + Fluconazole Mean	SEM
(6E,10E,14E,18E)-2,6,10,15,19,23-Hexamethyl-tetracosa-2,6,10,14,18,22-hexaene	Squalene	10,99	0,95	(-)	69	1,74	0,41	0,43	0,14	0,40	0,08	0,15	0,01
Ergosta-5,7,22- trien-3β-ol	Ergosterol	16,88	1,46	469	363	1,12	0,16	0,12	0,06	0,56	0,02	0,10	0,01
Ergosta-7-en-3b-ol	/	18,23	1,57	473	255	0,0064	0,0005	0,0009	0,0004	0,0022	0,0001	0,0005	0,0001
4,4,14-Trimethylcholesta-8,24(28)-dien-3β-ol	Lanosterol	18,82	1,62	499	393	0,15	0,02	0,24	0,03	0,05	0,01	0,13	0,04
14-Methylergosta-8,24(28)-dien-3β-ol	14-Methyl-fecosterol	17,84	1,54	485	379	0,016	0,003	0,022	0,006	0,003	0,001	0,008	0,001
14-Methylergosta-8,24(28)-dien-3b,6a-diol	/	18,65	1,61	573	467	0,00	0,00	0,24	0,08	0,00	0,00	0,11	0,02
Cholesta-8,24-dien-3β-ol	Zymosterol	16,56	1,43	457	69	0,0046	0,0002	0,0006	0,0001	0,0011	0,0001	0,0003	0,0001
4,4-Dimethylcholesta-8,24-dien-3β-ol	T-MAS	19,19	1,66	485	135	0,0161	0,0019	0,0041	0,0004	0,0131	0,0043	0,0019	0,0005
(22E)-Ergosta-5,7,9(11),22-tetraen-3?-ol		16,00	1,38		251	0,0026	0,0002	0,0036	0,0004	0,0017	0,0003	0,0017	0,0001
Sterol X	sterol 16-62	16,45	1,42		123	0,0020	0,0005	0,0120	0,0031	0,0004	0,0001	0,0053	0,0005
Sterol Y	sterol 17-57	17,38	1,50	469	469	0,0078	0,0017	0,0074	0,0011	0,0011	0,0003	0,0031	0,0005
Sterol Z	Compound D	23,91	2,06	469	469	0,0361	0,0204	0,0190	0,0118	0,0636	0,0427	0,0532	0,0346

IUPAC name	Trivial name	RT	RRT	Molecular ion TMS-derivative (g/mol)	Ion used for quantification (m/z)	Relative abundance RPMI pH 5.0 WT Mean	SEM	WT + Fluconazole Mean	SEM	*MGE1* Mean	SEM	*MGE1* + Fluconazole Mean	SEM
(6E,10E,14E,18E)-2,6,10,15,19,23-Hexamethyl-tetracosa-2,6,10,14,18,22-hexaene	Squalene	10,99	0,95	(-)	69	0,61	0,06	0,99	0,09	0,23	0,04	0,23	0,02
Ergosta-5,7,22- trien-3β-ol	Ergosterol	16,88	1,46	469	363	0,87	0,02	0,26	0,01	0,66	0,04	0,20	0,03
Ergosta-7-en-3b-ol	/	18,23	1,57	473	255	0,0062	0,0005	0,0009	0,0001	0,0029	0,0002	0,0005	0,0000
4,4,14-Trimethylcholesta-8,24(28)-dien-3β-ol	Lanosterol	18,82	1,62	499	393	0,11	0,00	0,34	0,02	0,06	0,01	0,15	0,02
14-Methylergosta-8,24(28)-dien-3β-ol	14-Methyl-fecosterol	17,84	1,54	485	379	0,006	0,001	0,030	0,001	0,003	0,001	0,011	0,001
14-Methylergosta-8,24(28)-dien-3b,6a-diol	/	18,65	1,61	573	467	0,00	0,00	0,45	0,06	0,00	0,00	0,13	0,03
Cholesta-8,24-dien-3β-ol	Zymosterol	16,56	1,43	457	69	0,0058	0,0013	0,0006	0,0000	0,0029	0,0005	0,0006	0,0002
4,4-Dimethylcholesta-8,24-dien-3β-ol	T-MAS	19,19	1,66	485	135	0,0303	0,0076	0,0070	0,0005	0,0223	0,0057	0,0023	0,0003
(22E)-Ergosta-5,7,9(11),22-tetraen-3?-ol		16,00	1,38		251	0,0016	0,0002	0,0025	0,0004	0,0019	0,0004	0,0011	0,0001
Sterol X	sterol 16-62	16,45	1,42		123	0,0012	0,0003	0,0171	0,0017	0,0013	0,0006	0,0064	0,0013
Sterol Y	sterol 17-57	17,38	1,50	469	469	0,0017	0,0006	0,0093	0,0010	0,0010	0,0003	0,0036	0,0006
Sterol Z	Compound D	23,91	2,06	469	469	0,0060	0,0014	0,0049	0,0005	0,0039	0,0005	0,0038	0,0009

IUPAC name	Trivial name	RT	RRT	Molecular ion TMS-derivative (g/mol)	Ion used for quantification (m/z)	Relative abundance RPMI pH 5.0 + 0.5% Acetate WT Mean	SEM	WT + Fluconazole Mean	SEM	*MGE1* Mean	SEM	MGE1 + Fluconazole Mean	SEM
(6E,10E,14E,18E)-2,6,10,15,19,23-Hexamethyl-tetracosa-2,6,10,14,18,22-hexaene	Squalene	10,99	0,95	(-)	69	0,67	0,15	0,30	0,03	0,09	0,02	0,085	0,003
Ergosta-5,7,22- trien-3β-ol	Ergosterol	16,88	1,46	469	363	0,48	0,05	0,12	0,02	0,15	0,02	0,070	0,003
Ergosta-7-en-3b-ol	/	18,23	1,57	473	255	0,0047	0,0009	0,0009	0,0001	0,0010	0,0002	0,0003	0,0000
4,4,14-Trimethylcholesta-8,24(28)-dien-3β-ol	Lanosterol	18,82	1,62	499	393	0,06	0,01	0,10	0,01	0,01	0,00	0,048	0,001
14-Methylergosta-8,24(28)-dien-3β-ol	14-Methyl-fecosterol	17,84	1,54	485	379	0,002	0,001	0,006	0,001	0,000	0,000	0,003	0,000
14-Methylergosta-8,24(28)-dien-3b,6a-diol	/	18,65	1,61	573	467	0,00	0,00	0,09	0,03	0,00	0,00	0,029	0,004
Cholesta-8,24-dien-3β-ol	Zymosterol	16,56	1,43	457	69	0,0037	0,0010	0,0004	0,0001	0,0009	0,0001	0,0001	0,0000
4,4-Dimethylcholesta-8,24-dien-3β-ol	T-MAS	19,19	1,66	485	135	0,0166	0,0011	0,0042	0,0004	0,0071	0,0007	0,0025	0,0002
(22E)-Ergosta-5,7,9(11),22-tetraen-3?-ol		16,00	1,38		251	0,0013	0,0001	0,0014	0,0002	0,0005	0,0001	0,0006	0,0001
Sterol X	sterol 16-62	16,45	1,42		123	0,0014	0,0005	0,0045	0,0009	0,0004	0,0001	0,0017	0,0001
Sterol Y	sterol 17-57	17,38	1,50	469	469	0,0008	0,0002	0,0014	0,0003	0,0001	0,0000	0,0004	0,0000
Sterol Z	Compound D	23,91	2,06	469	469	0,0023	0,0005	0,0021	0,0003	0,0014	0,0006	0,0027	0,0003

Supplementary Figure 2. Sterols detected by gas chromatography-mass spectrometry (GC-MS) in the WT and *MGE1* overexpression strains. Cells were grown in RPMI medium containing 0.2% glucose at pH 7.0, or pH 5.0, and/or containing 0.5% acetate, in the presence or absence of fluconazole for 24 h. The sterol composition of WT and *MGE1* strains was analyzed under all conditions. The abundance of each compound was calculated as the peak

area relative to cholestane (internal standard) based on CG-MS analysis of three biological replicates. RT, retention time; RRT, relative retention time.

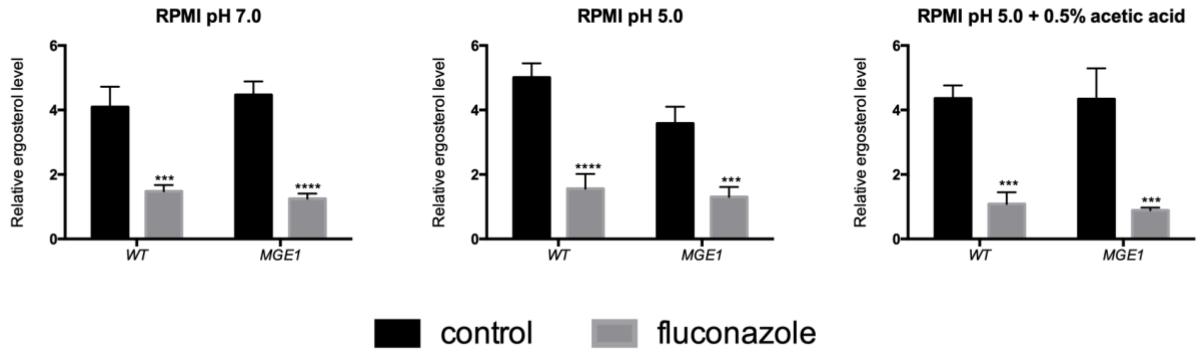

Supplementary Figure 3. Validation of GS-MS analysis according to the method described in Morio *et al.*, 2012.[80] Cells were grown in RPMI medium containing 0.2% glucose at pH 7.0, or pH 5.0, and/or containing 0.5% acetate, in the presence or absence of fluconazole for 24 h. Error bars show standard deviation. * $p < 0.05$, ** $p < 0.01$, *** $p < 0.001$, **** $p < 0.0001$ were considered statically significant relative to *C. glabrata* WT cells.

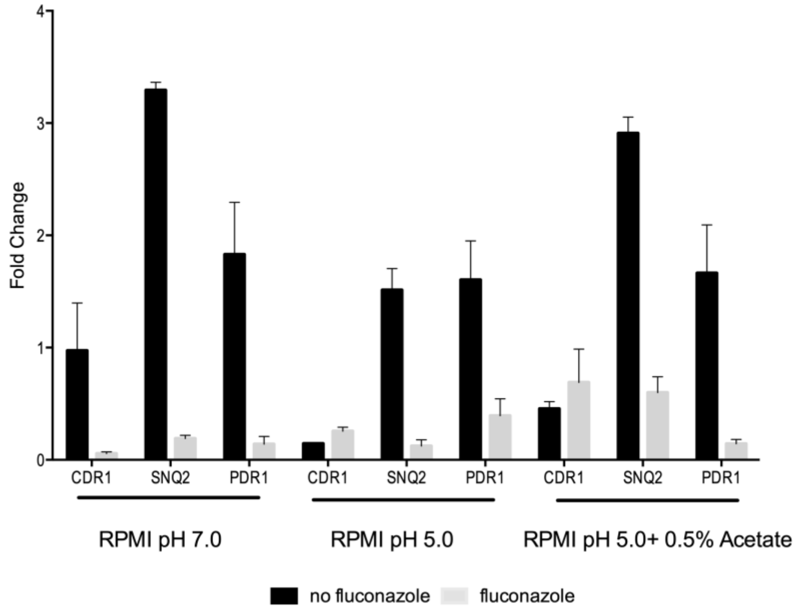

Supplementary Figure 4. Evaluation of the expression levels of three efflux pumps by qRT-PCR. *C. glabrata* biofilm cells were grown using glucose as sole carbon source (RPMI pH 7.0 or RPMI pH 5.0) or glucose and acetate (RPMI pH 5.0 + 0.5% Acetate), in the presence or

absence of fluconazole. Graphs show fold change expression of each gene compared to a housekeeping gene, *PGK1*.

Supplementary Table 1. Real-Time PCR conditions

Gene	Primer	Sequence	Product size (bp)	Tm (ºC)
CAT5	Forward	TATCCGTGTCGACCAAGC	192	54
	Reverse	AAGGGGGTCAGCAATGAT		
COQ6	Forward	CCACCAGAGCATTTCACC	169	55
	Reverse	GCTTTTCCAAATCGAGCA		
ERG9	Forward	TCCTCGGCGTAGTCTCTGAT	150	56
	Reverse	ATTGTCCTTGCAGGTTTTGG		
ERG11	Forward	CACCGGTTACACCGTCTTCT	145	55
	Reverse	CAGGACCTTGTCGTTACCGT		
FTR1	Forward	TCGCACTCGGGTTCTTTA	163	58
	Reverse	CGCAACATTGGAATACCC		
ATX1	Forward	AGAGGCGCTCGAAGGTGTGA	173	54
	Reverse	ACCCACGACATAGCGCAGGA		
CDR1	Forward	TTGTTGGTGTTCCTGGTGAA	142	58
	Reverse	ATGGACCATGCTGTTTGTGA		
SNQ2	Forward	CGATGCACCAACCAAGTATG	130	58
	Reverse	ACCACCGACAGTCATCAACA		
PDR1	Forward	CGGTGAGTTGGCCCTTACAA	171	58
	Reverse	TTTAATGTCGGCGGTTTCGC		
MGE1	Forward	GACGTTGAGAAGGCCAAGAG	80	58
	Reverse	CGCATGTCCAAAGTTATCCA		
PGK1	Forward	CAAACGGTGAAAGAAACGAGAA	100	58
	Reverse	CCGACACAGTCGTTCAAGAAAG		
